# Understanding spatiotemporal patterns of COVID-19 incidence in Portugal: A functional data analysis from August 2020 to March 2022

**DOI:** 10.1371/journal.pone.0297772

**Published:** 2024-02-01

**Authors:** Manuel Ribeiro, Leonardo Azevedo, André Peralta Santos, Pedro Pinto Leite, Maria João Pereira

**Affiliations:** 1 CERENA, DER, Instituto Superior Técnico, Universidade de Lisboa, Lisbon, Portugal; 2 Direção de Serviços de Informação e Análise, Direção-Geral da Saúde, Lisbon, Portugal; 3 NOVA National School of Public Health, Public Health Research Centre, Universidade NOVA de Lisboa, Lisbon, Portugal; Eduardo Mondlane University: Universidade Eduardo Mondlane, MOZAMBIQUE

## Abstract

During the SARS-CoV-2 pandemic, governments and public health authorities collected massive amounts of data on daily confirmed positive cases and incidence rates. These data sets provide relevant information to develop a scientific understanding of the pandemic’s spatiotemporal dynamics. At the same time, there is a lack of comprehensive approaches to describe and classify patterns underlying the dynamics of COVID-19 incidence across regions over time. This seriously constrains the potential benefits for public health authorities to understand spatiotemporal patterns of disease incidence that would allow for better risk communication strategies and improved assessment of mitigation policies efficacy. Within this context, we propose an exploratory statistical tool that combines functional data analysis with unsupervised learning algorithms to extract meaningful information about the main spatiotemporal patterns underlying COVID-19 incidence on mainland Portugal. We focus on the timeframe spanning from August 2020 to March 2022, considering data at the municipality level. First, we describe the temporal evolution of confirmed daily COVID-19 cases by municipality as a function of time, and outline the main temporal patterns of variability using a functional principal component analysis. Then, municipalities are classified according to their spatiotemporal similarities through hierarchical clustering adapted to spatially correlated functional data. Our findings reveal disparities in disease dynamics between northern and coastal municipalities versus those in the southern and hinterland. We also distinguish effects occurring during the 2020–2021 period from those in the 2021–2022 autumn-winter seasons. The results provide proof-of-concept that the proposed approach can be used to detect the main spatiotemporal patterns of disease incidence. The novel approach expands and enhances existing exploratory tools for spatiotemporal analysis of public health data.

## Introduction

Due to the rapid spread of SARS-CoV-2 worldwide, with the number of positive cases and affected countries growing exponentially, a pandemic was declared on 11 March 2020 [1]. From this time, more than 650 million cases have been confirmed (https://covid19.who.int/, last accessed Dec 27, 2022), forcing governments and researchers around the world to collect, analyse and interpret COVID-19 data to understand the pandemic’s evolution at different places and times. At the population level, where the epidemic occurs, several factors do promote the spread of infection, such as: climate [2, 3]; socio-economic conditions [4, 5]; and demographic characteristics (e.g., gender or age-structure). In addition, governmental interventions (e.g., social distancing, vaccination) are key in controlling and mitigating the spread of disease [6, 7]. As populations live in vast geographical domains with distinct dynamics, the distribution of COVID-19 incidence rates across time and space may differ substantially. At the same time, when modelling the spatiotemporal evolution of the disease, we should consider the fact that regions close to each other (culturally, economically, or geographically) tend to have similar COVID-19 incidence patterns since their populations tend to be exposed to similar conditions and lifestyles [8–11].

Due to this complex diversity of spatiotemporal patterns, it is difficult to describe and understand the underlying dynamics of COVID-19 incidence that are common across regions over time. Their spatiotemporal distribution has usually been recorded as time-series observed in different regions. Quantitative exploratory approaches using time-series data have been applied to model the temporal [12] and spatiotemporal [13] evolution of COVID-19 cases, mortality over different regions [14], to identify vaccine hesitancy clusters [15] or to extract mobility trends at different spatial scales [16]. These methods could be supplemented with exploratory data analysis tools (e.g., dimension reduction techniques or unsupervised classification methods) to detect patterns or to discriminate differentiating features [17, 18]. However, while these tools aim to describe and understand the spatiotemporal patterns of COVID-19 incidence, they do not explicitly consider the spatial correlations occurring over time. A comprehensive approach would explicitly combine the spatiotemporal distribution of COVID-19 incidence, along with exploratory analysis tools adapted to consider the spatiotemporal structure of the data. This would help to provide further understanding of spatiotemporal patterns of disease incidence, allowing public health authorities to make more informed decisions and adjust strategies accordingly.

Functional data analysis (FDA) transforms time-series observations into smoothing functions providing a model for the underlying process that gives rise to them. Despite being successfully used in many different scientific areas [19] such as biology [20], economics [21] and medicine [22], functional data analysis is still relatively recent in the field of public health. Classical statistical tools like principal component analysis or hierarchical clustering methods have been adapted to functional data, and offer an appealing option for spatiotemporal modelling. These methods have been used for exploratory analysis, description, and classification of COVID-19 mortality, providing useful insights into patterns of mortality across different regions of Italy [23], by COVID-19. Still, these exploratory tools rely on the assumption of independence between functional data, and do not consider the spatial distribution observed in the data. In the last decade, promising approaches have emerged in applied sciences (e.g., meteorology) to adapt exploratory tools considering the spatial structure of functional data [24].

In this study, we propose a novel approach to unveil the main spatiotemporal patterns underlying COVID-19 incidence on mainland Portugal. The method is based on a combination of functional data analysis and unsupervised learning algorithms (i.e., functional principal component analysis and hierarchical classification) to extract the main patterns of disease incidence over time, while simultaneously considering the spatial correlations of incidence rates across regions. By including spatial information in this approach, the spatial dependence of different regions can be explicitly accounted for, providing a more comprehensive tool to describe the major regional patterns of incidence over time. The approach is illustrated by the analysis of the daily number of newly COVID-19 cases reported for 570 consecutive days at the municipality level on mainland Portugal. The period analysed was characterized by a bundle of Non-Pharmacological Interventions (NPI), where restrictive measures were balanced against the impact on populations’ health, social and economic stability. We first detail the proposed methods, followed by their application to mainland Portugal. The results are then discussed, before presenting the main conclusion of this study.

## Materials and methods

Data was collected within the national SCOPE (Spatial Data Sciences for COVID-19 Pandemic) research project, which aims to deliver novel modelling tools to support decision-making in the COVID-19 pandemic [25, 26]. As part of the project, we analysed, from a functional data perspective, 278 time-series with daily COVID-19 incidence data collected from all mainland Portuguese municipalities (i.e., 278 functions).

The proposed modelling method assumes that time-series data are derived from underlying processes that can be modelled with a set of smoothing functions (curves). These curves are combined with a functional principal component analysis (FPCA) and hierarchical classification of spatially correlated data to extract spatial patterns of COVID-19 from the set of incidence curves. With FPCA, we analysed the dominant types of variation among municipality curves, while their patterns were classified using a dissimilarity matrix which considers spatial covariance among functional data. Each of these steps is described in the following subsections. All modelling and data visualization was performed in open-source software R [27] with packages fda [28] for functional data and functional principal component analysis, geofd [29] for hierarchical classification of spatially correlated functional data and ggplot2 [30] for data visualization.

### Study area

**Fig 1:**
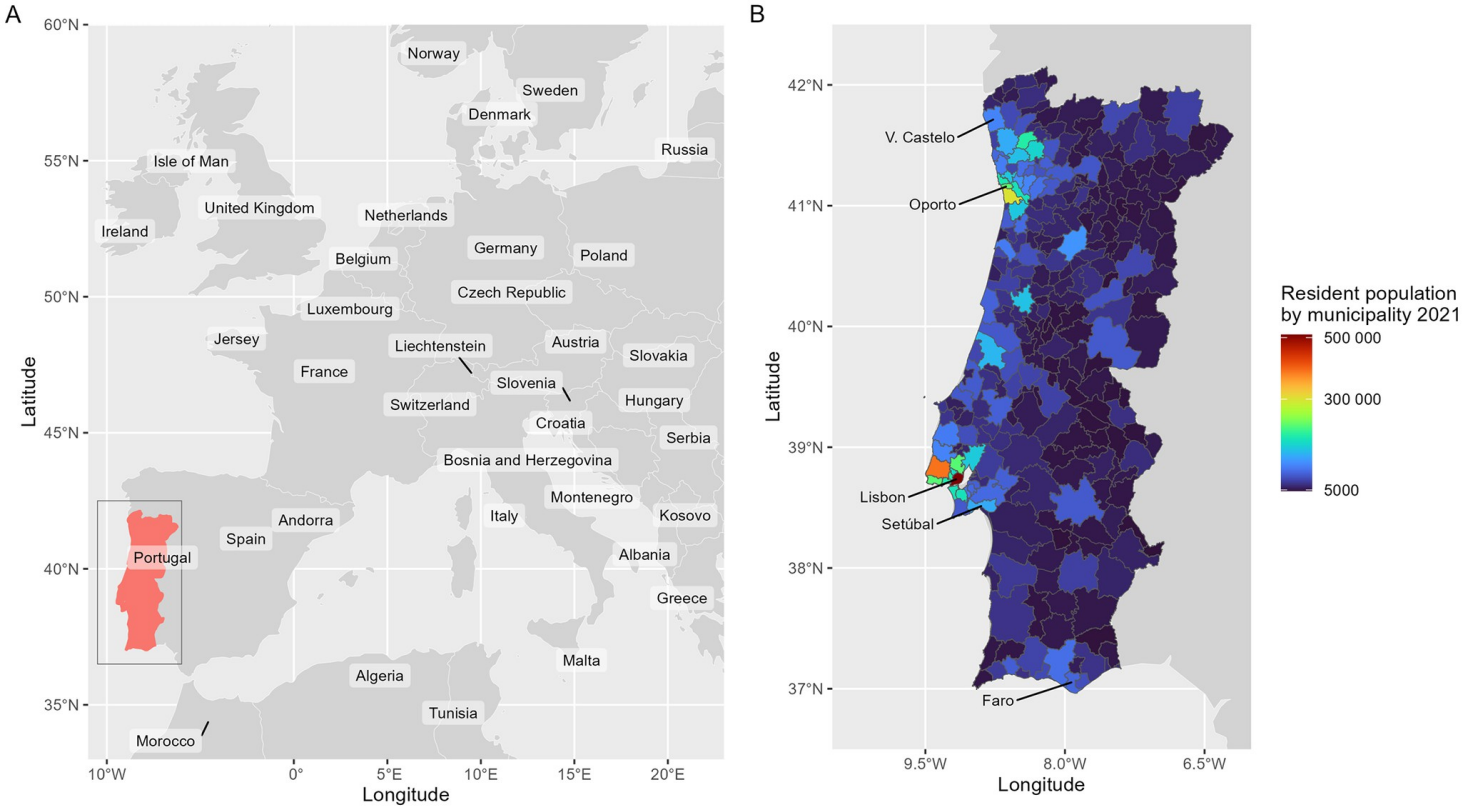
Geographical location of the study area with population by municipality. Map of Europe with the location of mainland Portugal (A) and mainland Portugal with population distribution by municipality (B). All base maps used are in public domain, courtesy of Natural Earth (http://www.naturalearthdata.com/) and the Portuguese Direção-Geral do Território (https://www.dgterritorio.gov.pt/). Population data is sourced by the Instituto Nacional de Estatística–Portugal (Statistics Portugal) and can be used in accordance with the Creative Commons CC BY Attribution 4.0 license.

The country of Portugal includes the mainland, located in southern Europe, and the archipelagos of Azores and Madeira, in the Atlantic Ocean. We restrict our study area to mainland as the archipelagos of Azores and Madeira have their own healthcare reporting system. Mainland Portugal is located on the Iberian Peninsula, and is bordered by Spain (to the east and north) and the Atlantic Ocean (to the west and south), covering a total area of 89102 square kilometres (Fig 1).

The resident population in year 2021 was 9.8M habitants [31]. The population density is higher along the western coastline, especially between Setúbal and Viana do Castelo, and along the southern coastline, especially in the Faro region and surrounding areas. The metropolitan areas of Lisbon and Porto are the most populous regions (4.6M population). In the north, centre and hinterland, particularly in the region south of Lisbon, there are several medium and small-sized urban areas with low population sizes.

### Data collection

The time-series reporting the daily number of newly confirmed COVID-19 cases between 14 August 2020, and 6 March 2022 (*T* = 570 days), at the municipality level (N = 278 municipalities) on mainland Portugal, was obtained from the Portuguese Epidemiological Surveillance System (SINAVE) database and provided by DGS. While our analysis starts at the end of the first epidemic wave (August 2020), the original dataset included all confirmed cases reported between 2 March 2020 (date of the first confirmed COVID-19 cases in Portugal) and 6 March 2022. During the first wave, Portugal implemented a successful nationwide lockdown, as well as a combination of non-pharmaceutical interventions [32], leading to a slow evolution of the pandemic [33, 34] until September 2020. During this period, most of municipalities with low population densities notified zero or near-zero daily numbers of new cases. To avoid analysing periods of sparse data, we began our analysis in August 2020 (final part of first epidemic wave) close to the onset of the second wave.

The estimated population at risk by municipality was supplied by Statistics Portugal (https://www.ine.pt/). The daily indicator used for analysis was the 14-day cumulative incidence rate of COVID-19 by municipality, resulting from the quotient of the number of new confirmed COVID-19 cases reported (numerator) in the previous 14 days and the population at risk (denominator) per 100,000 people (hereinafter referred to as "daily incidence rates").

We analysed *N* time-series of daily incidence rates (*r*) observed at *T* time points, which can be represented as ${\left\{r_{i}\left(t_{j}\right):j=1,\ldots ,T\right\}}_{i=1}^{N}$ where *r*_*i*_(*t*_*j*_) represents incidence rate at day *j* (with *j* = 1,…, *T*) in municipality *i* (*i* = 1,…, *N*).

### Ethics declaration

The Portuguese Directorate-General of Health (DGS) provided the data to conduct this study in accordance with the Article 39 of Legislative Decree 2-B/2020 of 2 April 2020. The COVID-19 data used in this study are anonymized, which implies the removal of any personal data; therefore, informed consent was not required. The analysis was performed on aggregate-level data in accordance with relevant guidelines and regulations.

### Functional data analysis

A functional data analysis (FDA) was used to model and describe COVID-19 incidence rates by municipality as a function of time. Under this approach, discrete data are converted into smooth continuous curves assuming the existence of underlying functions defined over a compact (continuous) temporal domain, from which time-series data are observed. The approach provides a parsimonious representation of data [35] and a natural way to thinking through curve smoothing procedures that allows for data noise reduction [36, 37]. Different basis functions (e.g., splines, polynomials, wavelets, or Fourier) are used for smoothing, and are chosen depending on the kinds of data available. For example, splines are better suited to non-periodic, noisy data, and Fourier bases for periodic data. The underlying smoothing function for the *i*-th municipality can be defined in the compact interval {*r*_*i*_(*t*): *t* ∈ [0,*T*]} and assumed to belong to a Hilbert space [38] by means of a basis system (i.e., a linear combination of basis functions):

$$r_{i}\left(t\right)=\sum_{j=1}^{J}c_{ji}\Phi_{j}\left(t\right)=c_{i}^{T}\Phi \left(t\right),$$ (1)

where **Φ**(*t*) is a vector of *J* standard basis functions, and **c**_***i***_ is a vector of length *J*, with the coefficients used to build functional data *r*_*i*_(*t*). The vector **c**_***i***_ can be estimated by minimizing the sum of squared errors (i.e., least squares estimator), with a roughness penalty (regularization) that looks for an optimal balance between data fit and the original curves and smoothness. The degree of smoothness depends on the number of *J* basis functions considered. On one hand, if the number *J* is large, the sum of squared errors will be small, but the risk of overfitting increases. On the other hand, if *J* is small, relevant information about local features might be missed. In practice, the usual approach for setting *J* is determined by trial and error. According to the characteristics of the available time-series data, we have chosen to use a cubic B-spline basis every 12 days, resulting in a total of 48 knots equally spaced, to approximate the functional form. A smoothing parameter of 0.25 was applied to the estimated functional parameters, which minimized the average generalized cross-validation error across the 278 incidence rate curves.

### Functional principal component analysis

In this study, we used a functional principal component analysis (FPCA) to explore the variability within COVID-19 incidence curves. FPCA expands the application of classical principal component analysis (PCA), now in the context of functional data analysis [37]. Just like in PCA, FPCA looks for the dominant types of variation (maximizing variability, after subtracting the mean from each observation), by finding the decomposition of the covariance function that maximizes variance with a minimal number of (functional) principal components. Mean corrected curves, *r*_*i*_(*t*)*, are obtained after adjusting for the (pointwise) estimated mean curve $\bar{r}(t)=\frac{1}{n}\sum_{i=1}^{N}r_{i}(t)$:

$${r_{i}(t)}^{*}=r_{i}\left(t\right)-\;\bar{r}\left(t\right).$$ (2)

The covariance function, *v*(*t*,*s*), can be estimated by discretising *r*_*i*_(*t*)* into a regular grid of equally spaced values in the interval *t* ∈ [0,*T*] to form a data matrix suited to compute the covariance as:

$$v\left(t,s\right)=\frac{1}{N-1}\sum_{i=1}^{N}{r_{i}\left(t\right)}^{*}{r_{i}\left(s\right)}^{*}.$$ (3)

Once the decomposition is solved, eigenvalues and eigenfunctions are obtained, and new functional components can be interpreted as different types of variation; the implementation details of FPCA can be found in Ramsay and Silverman [37].

The functional principal component scores are linear combinations of functional data, *r*_*i*_(*t*)*, where eigenfunctions are their coefficients. For municipality *i* and principal component *α*, the functional principal score, *z*_*iα*_ is given by the integral:

$$z_{i\alpha }=\int \xi_{\alpha }(t){r_{i}(t)}^{*}dt,$$ (4)

where *ξ*_*α*_(*t*) is the eigenfunction associated with *α*.

Just like in classical PCA, the first principal components form a subspace representing the main types of variation found in the original data. Assuming that the first components provide a satisfactory approximation, they allow new insights and clearer interpretations about the covariance structure among COVID-19 incidence curves at any point in time. Once again, the strategies applied in classical PCA for selecting the main types of variation to interpret can also be applied in FPCA (e.g., scree plot or the proportion of variability explained). In our application example, we have chosen to consider the first three components, since they explained more than 70% of overall variability.

### Clustering spatial functional data

Hierarchical clustering is traditionally used in multivariate data analysis for unsupervised classification. Here we used an extension of the algorithm appropriate for dealing with spatially correlated functional data. As in the classical approach, hierarchical clustering uses agglomerative or divisive strategies [39] and a dissimilarity measure between functional data, which informs which clusters should be combined [40]. In this study, Euclidean distances (L2-norm) are used to build a dissimilarity matrix where the dissimilarity/distance between two curves *r*_*i*_(*t*)* and *r*_*j*_(*t*)* is given by:

$$d_{ij}=\sqrt[{2}]{\int_{a}^{b}{\left[{r_{i}(t)}^{*}-{r_{j}(t)}^{*}\right]}^{2}dt}$$ (5)

To deal with spatial dependency, we used geostatistical methods to cluster contiguous municipalities with similar curve patterns [35]. The trace-variogram is an adaptation of the classical variogram [41] and was used to capture the spatial dependence between functions, i.e., to measure an average (expected) increase in functional data value for a given increase in the geographical domain:

$$\gamma \left(h\right)=\frac{1}{2}E\left[\int_{a}^{b}{\left[{r_{i}\left(t\right)}^{*}-{r_{j}\left(t\right)}^{*}\right]}^{2}dt\right]\;\text{with}\;h=\left\|{r_{i}}^{*}-{r_{j}}^{*}\right\|.$$ (6)

Municipalities are geographically referenced by the cartesian coordinates of their population-weighted centroids. Once the trace-variogram was estimated, we could fit a theoretical model (e.g., exponential, spherical or gaussian variogram model) to the experimental trace-variogram to characterize the spatial dependency between curves.

The most suitable variogram was chosen to weight the dissimilarity matrix of functional data, as follows:

$$d_{ij}^{w}=d_{ij}*\gamma \left(h\right).$$ (7)

In this study, we clustered municipalities using 3 functional principal components. The dissimilarity matrix was weighted by an exponential variogram model given by:

$$\gamma \left(h\right)=c_{0}+c_{1}\left[\text{exp}\;\left(\frac{h}{a}\right)\right]$$ (8)

The exponential model parameters of partial sill (*c*_1_), nugget-effect (*c*_0_), and range (*a*) are estimated by non-linear least squares, and refer respectively to the parts of variability in data with and without spatial correlation, and the distance above which spatial correlation can be considered to be negligible.

## Results

**Fig 2:**
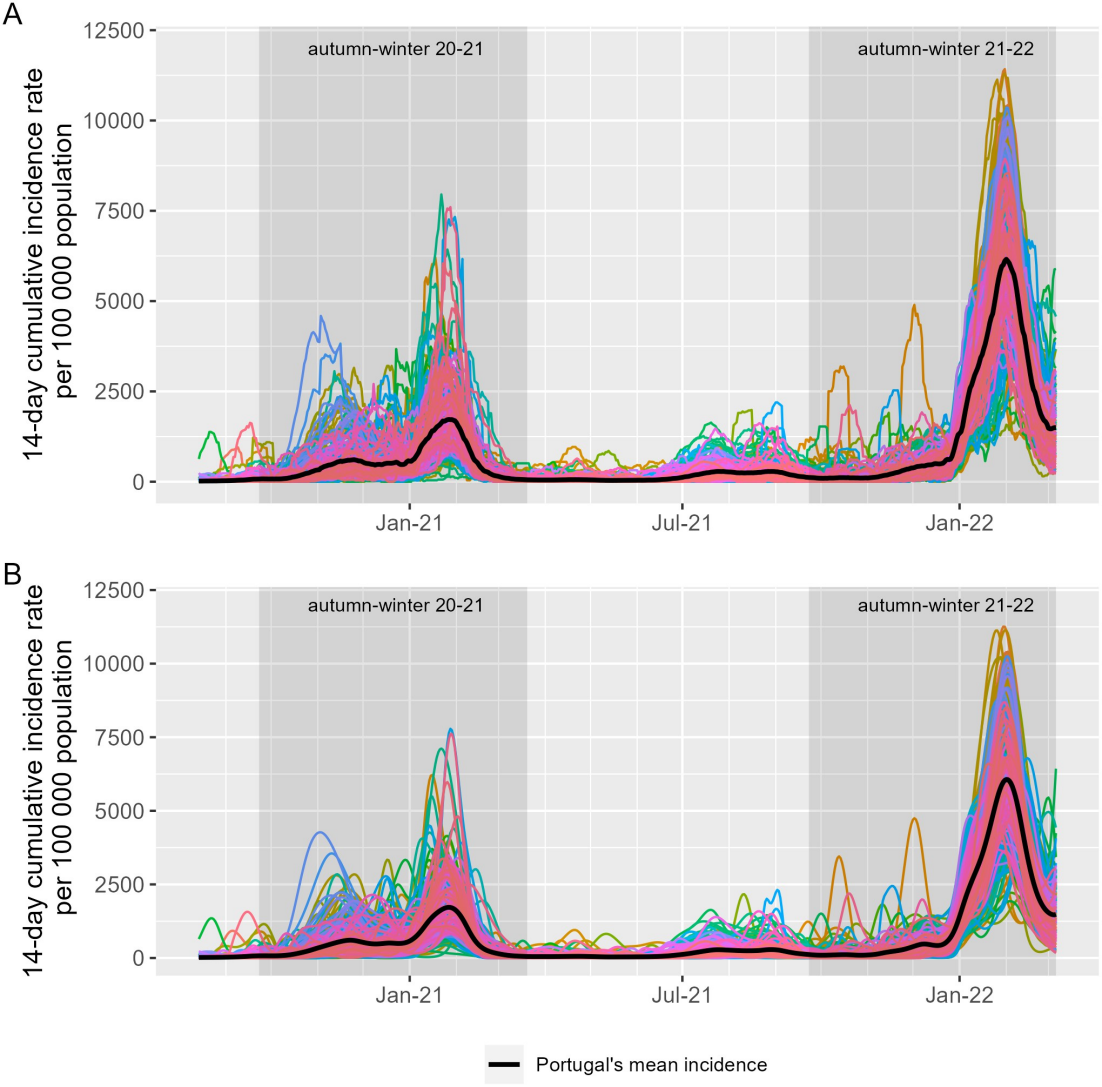
COVID-19 time series data before and after smoothing. COVID-19 time-series of the 278 municipalities during the period considered (from 14 August 2020 to 6 March 2022) (A), and the corresponding functional data sets (B). In A and B, each colour represents one of the 278 municipalities; the black curve represents the overall mean Portuguese incidence rate.

In the autumn-winter periods (i.e., between September and March, especially during the 2021–2022 season), incidence rates exhibited higher variability in amplitude (curve heights) when compared to other seasons (Fig 2A). The original time-series data were transformed into functional data composed of 278 curves (as in Eq 1), each corresponding to 570 days of incidence rates per municipality. Bringing the original data into functional form provided a smoother representation of the data (Fig 2B).

**Fig 3:**
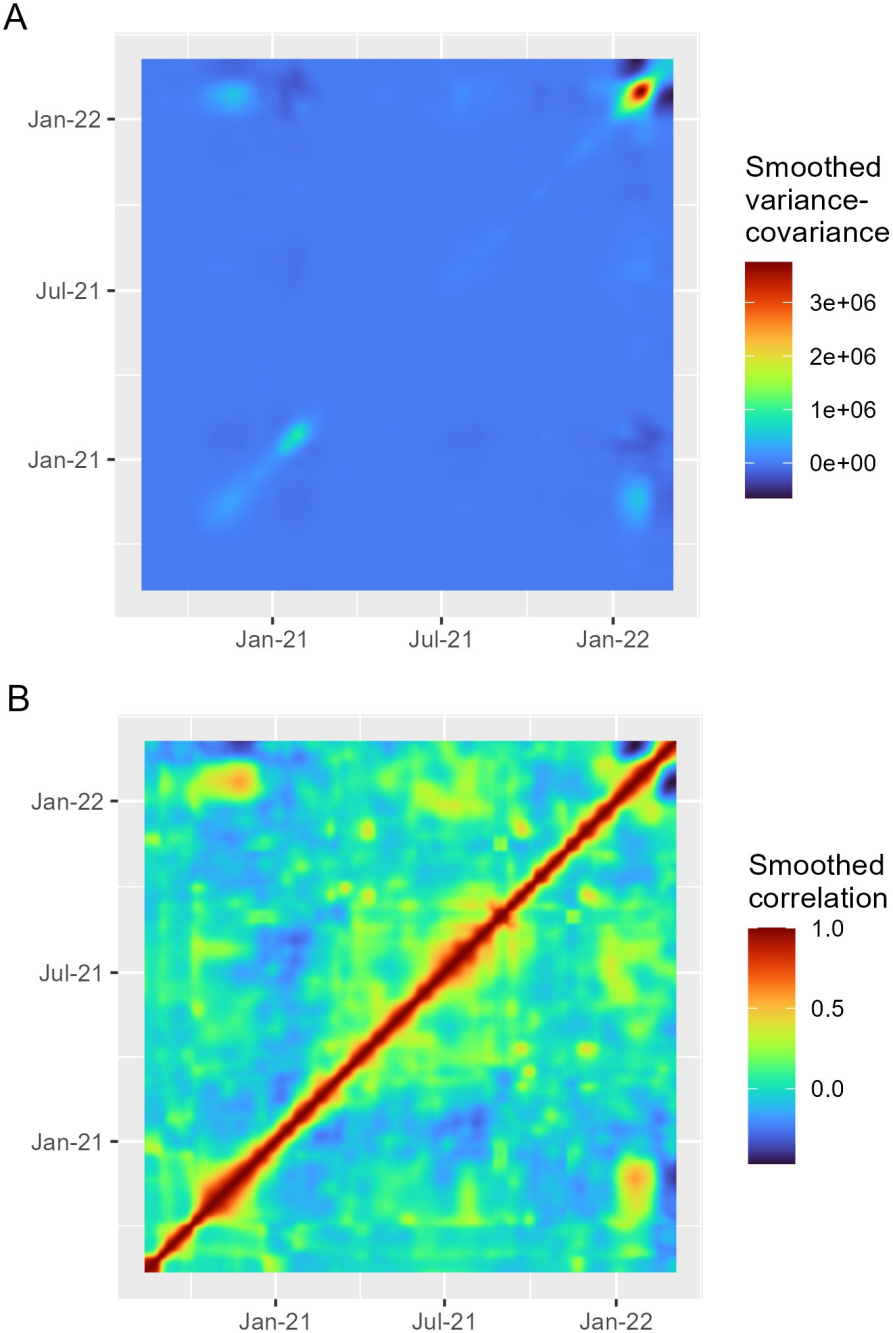
Variance-covariance and correlation surfaces of COVID-19 functional data between August 2020 and March 2022. Smoothed variance-covariance (A) and smoothed correlation (B) maps between pairs of days. The colour gradient highlights temporal periods with higher covariance/correlation values.

The smoothed variance-covariance (computed according to Eq 3) plot illustrates the nature of data variability after the transformations (Fig 3A). The diagonal ridge that crosses the graph refers to the daily variance of the functional data, evolving from 14 August 2020 to 6 March 2022. Higher variability can be seen in winter periods, especially in 2022, when variability reaches the highest values (between January and February). The smoothed correlation (Fig 3B) plot refers to a standardized version of the variance-covariance plot. We do observe that not only incidence correlations are higher at closer days, but also they tend to drop more rapidly in winter periods.

**Fig 4:**
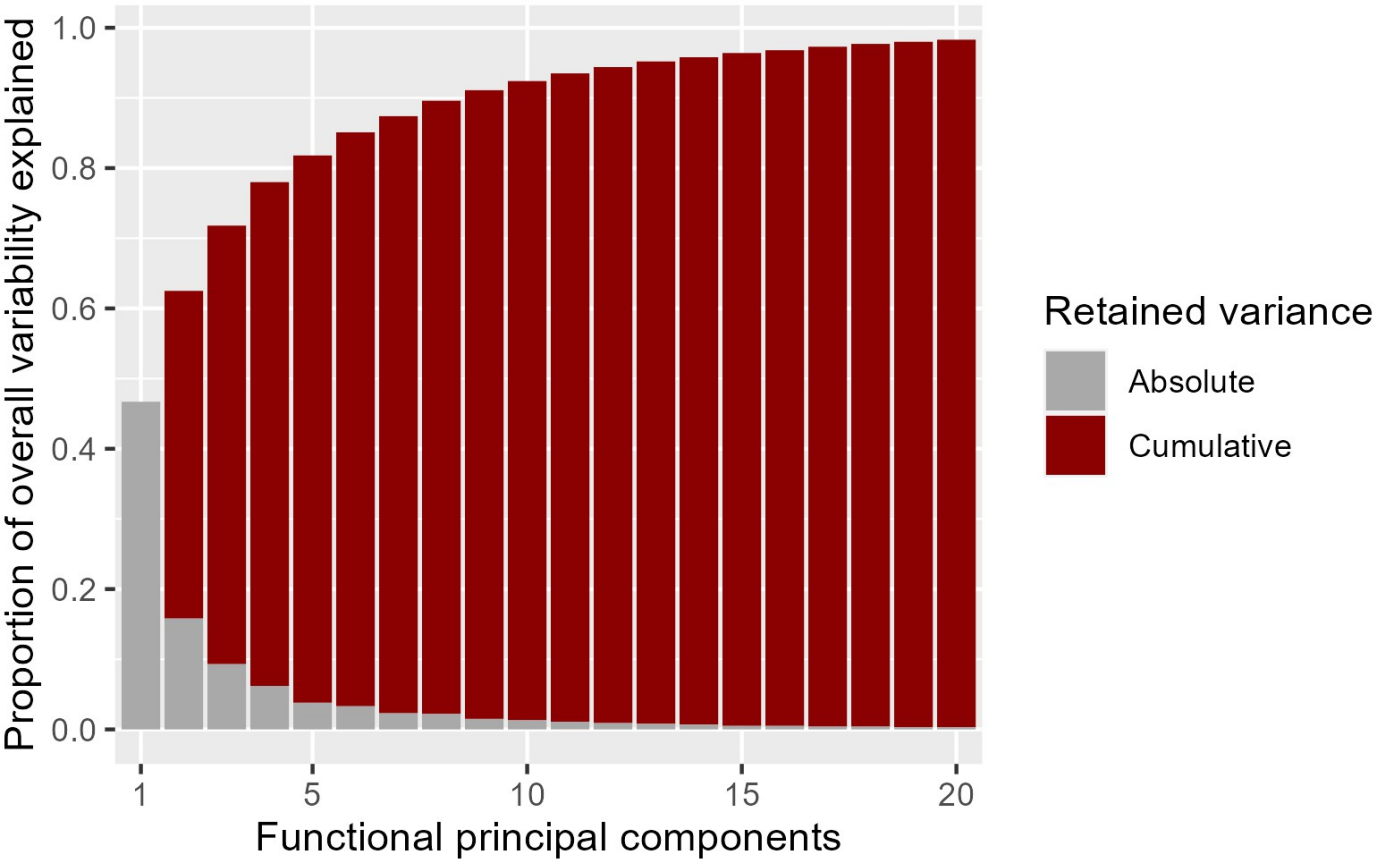
Scree-plot of the variance retained by the first 20 functional principal components. The scree plot shows the proportion of overall variability retained by each of the functional principal component curves (absolute variance) and the cumulative variability proportion retained by them (the cumulative variance is obtained by adding successive absolute variance proportions). Over 70% of overall variability is explained by the first 3 principal component curves.

To find the dominant types of variation in the functional data, we first centred the curves (mean adjustment) and computed principal components. The scree plot (Fig 4) illustrates the percentage of (absolute and cumulative) variance retained by the first 20 functional principal components. Over 70% of the overall variability observed was explained by the first 3 principal components.

**Fig 5:**
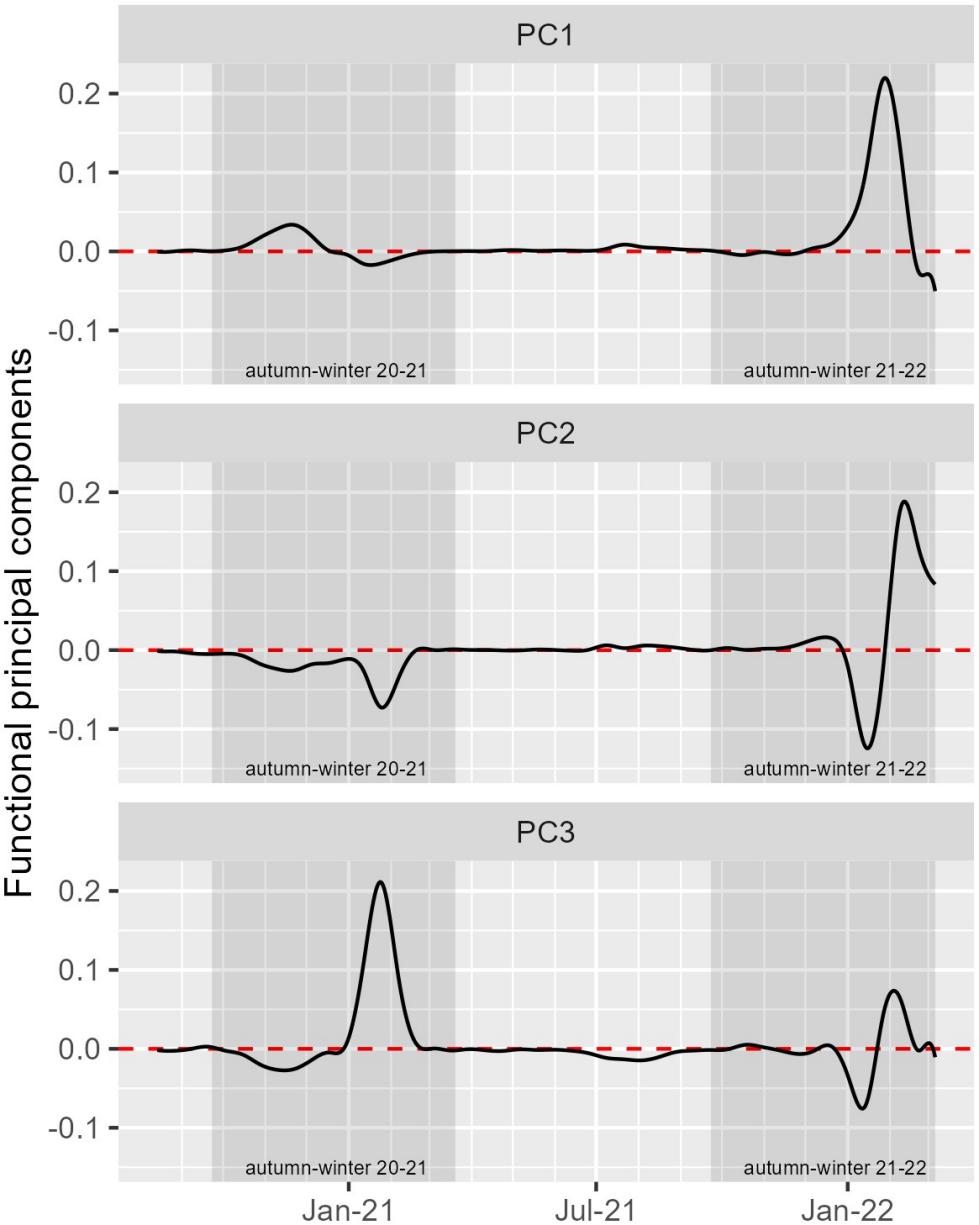
First three principal component curves for COVID-19 functional data. The first principal component (PC) retained 47% of overall variability, while the second and third accounted for 16% and 9%, respectively. The cumulative proportion of variation explained for the first three components is 72%.

The results obtained for the first three functional principal components (accounting for 72% of overall variability) are illustrated in Fig 5, as they represent the main types of variation observed in the data. These principal component curves reflect the main types of variation found in incidence, and allow us to identify the key features separating them over the time.

The first functional principal component (PC) represents 47% of overall variability, and determines the main type of variation shared by the 278 incidence curves. It reflects the major trends in the curves, as seen in Fig 2B, with higher incidence amplitudes in the autumn-winter seasons and more stable evolution in the spring-summer seasons. We may refer to this component as the “global effect”. Looking at the first PC curve, we can see four main phases of pandemic evolution: the first phase (August to October 2020) shows low differences in pandemic evolution across municipalities, while in the second (October 2020 to January 2021), differences between curves increased and reached a maximum around mid-November 2020 and, after that, began to decrease until mid-January 2021. The third phase (January 2021 to December 2021) is again characterized by low differences across municipalities. In the last phase (December 2021 to February 2022), considerable differences in incidence curves occurred across municipalities, as the pandemic curve increased severely and peaked in February 2022.

The second functional PC retained 16% of overall variability. The curves sharing this type of variation contributed to the decrease in the average incidence observed in the autumn-winter 2020–2021 period and between Christmas 2021 until the end of January 2022, ending up with a severe increase, similar to the first functional component, but reaching the peak slightly later (mid-February). The municipalities with high negative scores in this component were the most relevant contributors towards lowering the mean incidence rate observed in the referred periods. This functional PC will be referred as the “preventive measures effect”.

The third functional component accounts for 9% of overall variability, and corresponds to the increase of incidence caused by curves with high incidence rates in the winter periods, especially in 2020. This functional PC was negative until December 2020, and since then became positive, continuing to increase until reaching the peak on late-January 2021. After that, the curve started decreasing, but stayed positive until the end of February 2021. Over the autumn-winter period of 2021–2022, it presents a similar pattern to the second functional component, except for a smaller amplitude. This component will be referred as the “outbreak effect”. The remaining components represent small amounts of variation within the original 278 incidence curves (e.g., 6% and 4% for the fourth and fifth components, respectively), and are more difficult to interpret. Consequently, from here forward, the results to be presented are restricted to the first three principal components.

From a spatial perspective, it is interesting to visualize the functional PC scores, since they may contribute towards finding geographical patterns, clusters or outliers.

**Fig 6:**
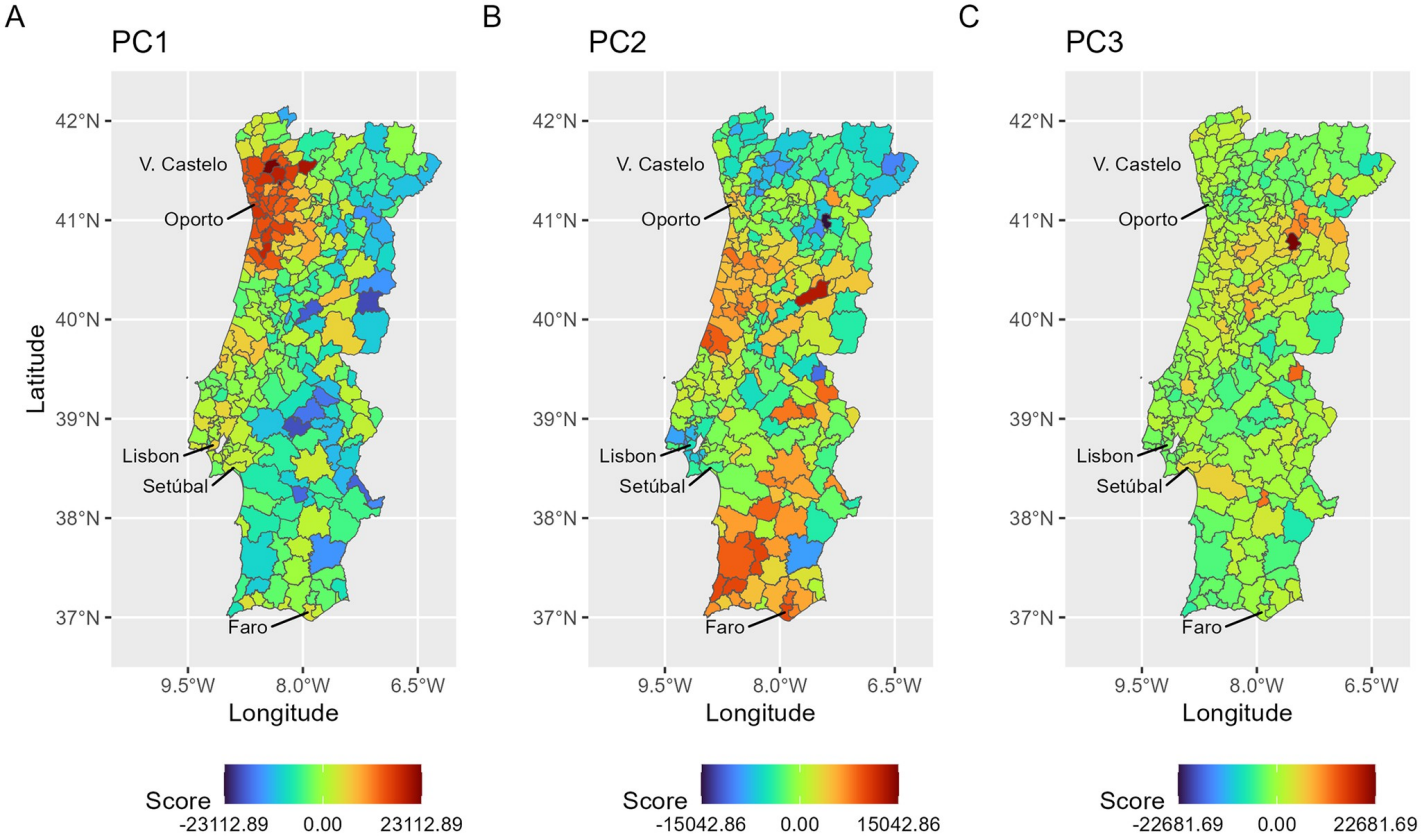
Spatial distribution of municipality scores in functional PC1 (A), PC2 (B) and PC3 (C). In each functional PC, the score of one municipality corresponds to the importance of that municipality curve to that functional PC. For example, in the first PC map (Fig 6A), we can see a cluster of municipalities located in the northern coast (Porto region) with the highest scores, meaning that their curves were the main contributors towards the variability retained in functional PC1. The official administrative base map of Portugal is in public domain, courtesy of the Portuguese Direção-Geral do Território (https://www.dgterritorio.gov.pt/).

For example, in the first PC (interpreted as the “global effect”), it is clear that the highest scores correspond to municipalities clustered along the northern coast and adjacent regions, where municipalities tend to be small and close to each other (Fig 6A). Municipalities from these regions were the most relevant contributors towards increasing the average incidence curve, since they reported high incidence rates over the autumn-winter seasons. On the other hand, the second functional PC (interpreted as the “preventive measures effect”) showed the highest negative scores in municipalities dispersed along Portugal’s hinterland, where municipalities are characterized by having sparse populations and separated by longer distances, plus the municipalities from the region of Lisbon, which were under severe restrictive measures during the pandemic (Fig 6B). In this case, negative scores are linked to municipalities that contributed towards lower average incidence rates during autumn-winter seasons, and positive scores to municipalities contributing towards the opposite direction. In fact, during the autumn-winter of 2020–2021, Portugal set tiered lockdowns (November 2020) and a nationwide lockdown with and without school closure (January 2021), with the Lisbon and Northern regions having higher proportions of municipalities under more restrictive measures [32]. In contrast, lower seroprevalence rates of SARS-CoV-2 antibodies were reported in the Algarve region (Southern region) following a large-scale vaccination campaign [42], suggesting a lower engagement of local populations in preventive behaviours. In the third component (Fig 6C), interpreted as the “outbreak effect”, the municipalities with high positive scores are spread across the country in places where population sizes are small, and where disease outbreaks (which occurred mainly in nursery houses) were reported.

**Fig 7:**
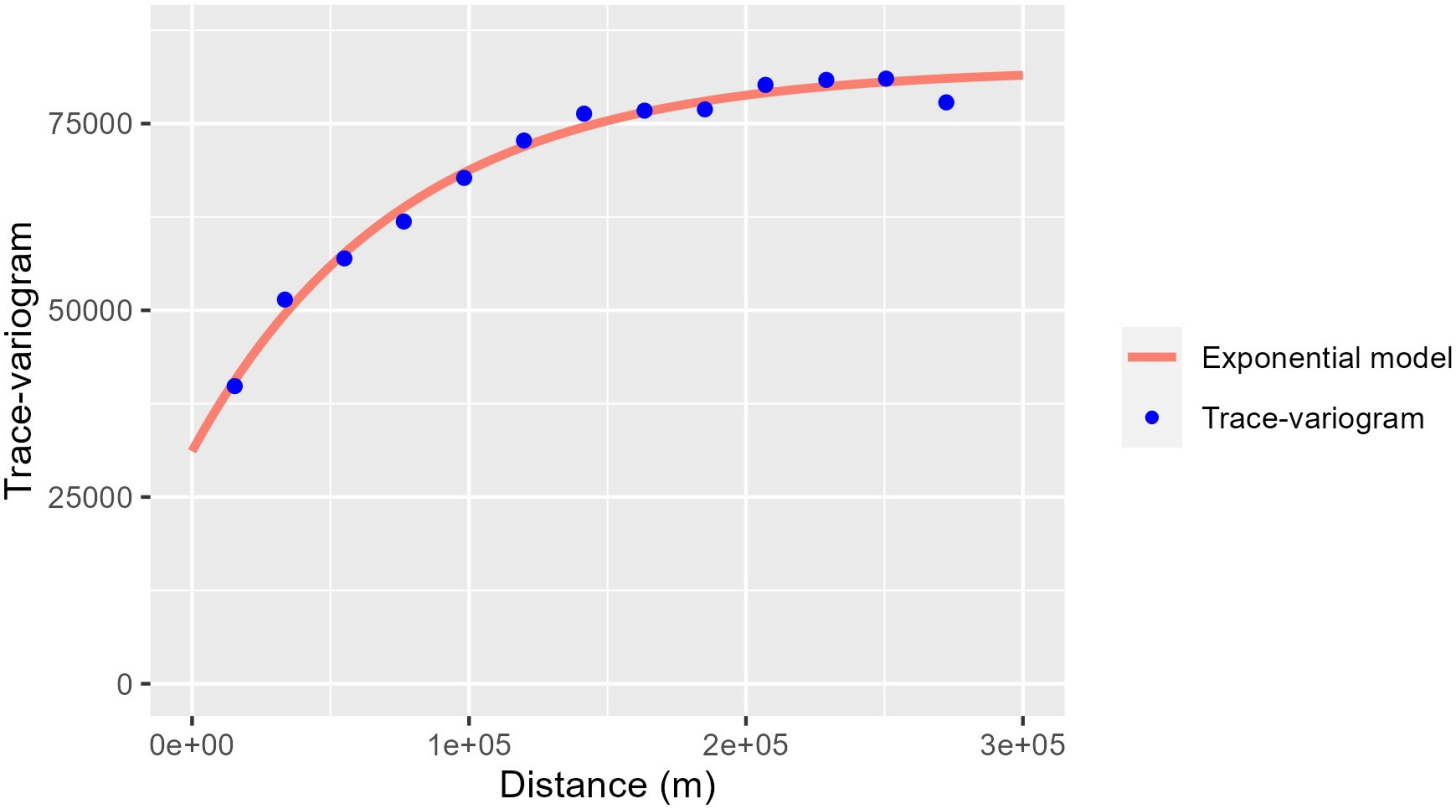
Trace-variogram and variogram fit to the COVID-19 functional dataset. The exponential variogram model was fit to trace-variogram estimates (blue filled circles) used to weight the dissimilarity matrix in hierarchical clustering (as in Eq 7). The x-axis refers to the Euclidean distance between pairs of municipalities, while the y-axis refers to the trace-variogram function values. The nugget-effect is the y-intercept. As distance increases, the values increase asymptotically up to the sill (nugget-effect + partial sill), after which the municipalities are considered to be no longer correlated.

To simplify the interpretation of principal component scores, we further classified municipalities into homogenous groups using the hierarchical classification algorithm as described above. To weight the dissimilarity matrix, we used an exponential variogram model (as in Eq 8) with an estimated nugget-effect of 31131 (38%), a partial sill equal to 51313 (62%) (units: squared 14-day cumulative incidence rate) and an estimated range parameter of 227 kilometres (Fig 7).

**Fig 8:**
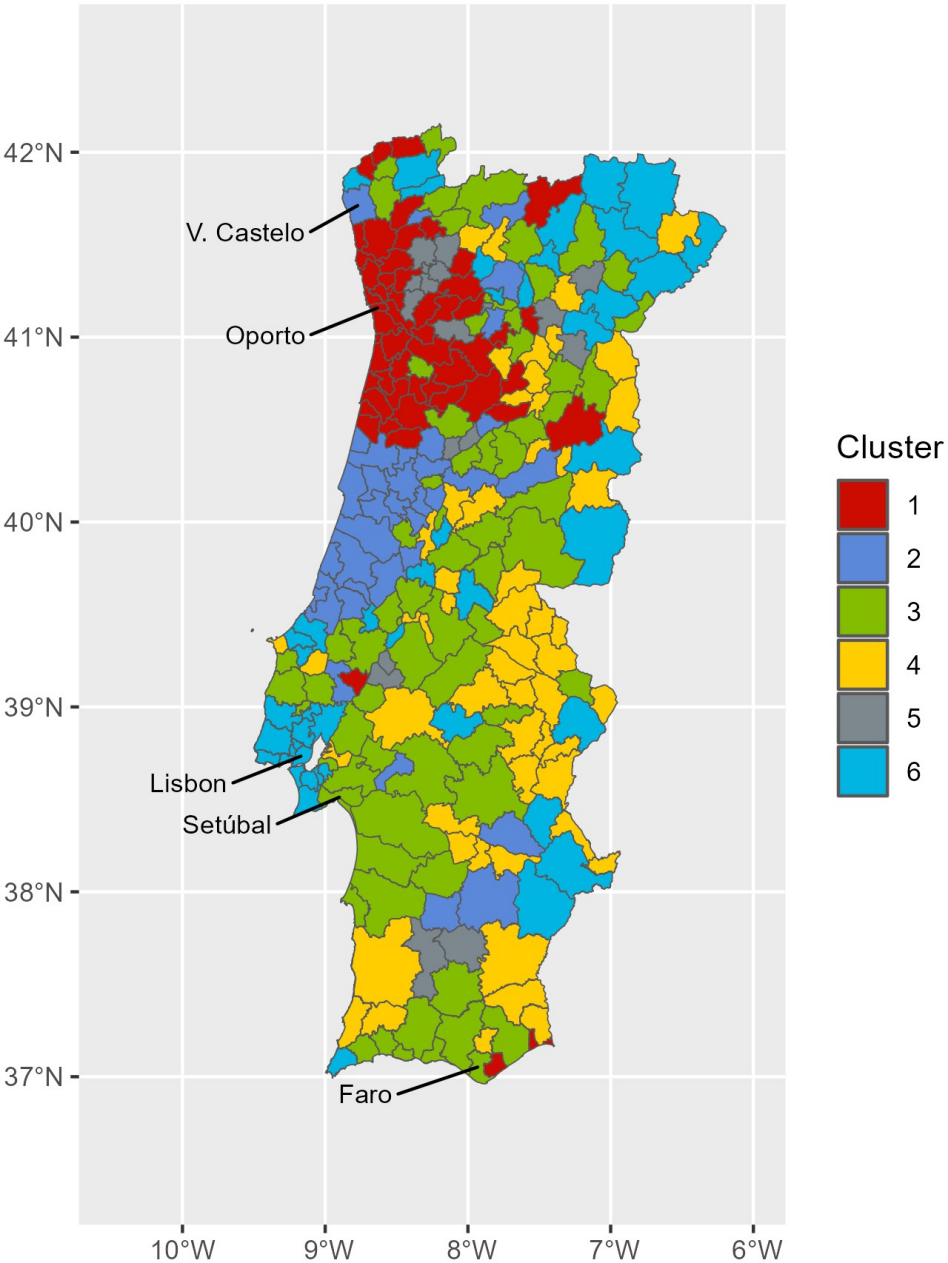
Spatial clusters of COVID-19 incidence in Portugal between August 2020 and March 2022. Spatial distribution of the Portuguese municipalities classified according to the results from hierarchical clustering with spatially correlated functional data. The number of municipalities classified in each cluster is as follows: Cluster 1 (red), 55; Cluster 2 (dark blue), 35; Cluster 3 (green), 69; Cluster 4 (yellow), 55; Cluster 5 (grey), 18; Cluster 6 (light blue), 46. The official administrative base map of Portugal is in public domain, courtesy of the Portuguese Direção-Geral do Território (https://www.dgterritorio.gov.pt/).

The estimated parameters indicate that functional data from municipalities separated up to 227 km are still correlated, but almost two fifths of the variability (38%) is not explained by the distances separating them (i.e., there is no spatial pattern). The dissimilarity matrix used is based on the Euclidean distances among the coefficients of the cubic-spline basis functions, while the ward-D2 linkage is applied as the agglomeration method. The dendrogram (not presented here) obtained was split in different ways to find an interpretable number of partitions. The tree cutting procedure (i.e., pruning) was guided by a visual inspection of results, and attempted to find an optimal number of partitions neither too small to identify any spatial clustering nor too big to miss the effect of spatial dependence found in the trace-variogram of functional data. As a result, the final dendrogram was cut into six groups, or clusters, where it is possible to detect spatially correlated patterns of municipalities groups according to the clusters assigned (Fig 8).

**Fig 9:**
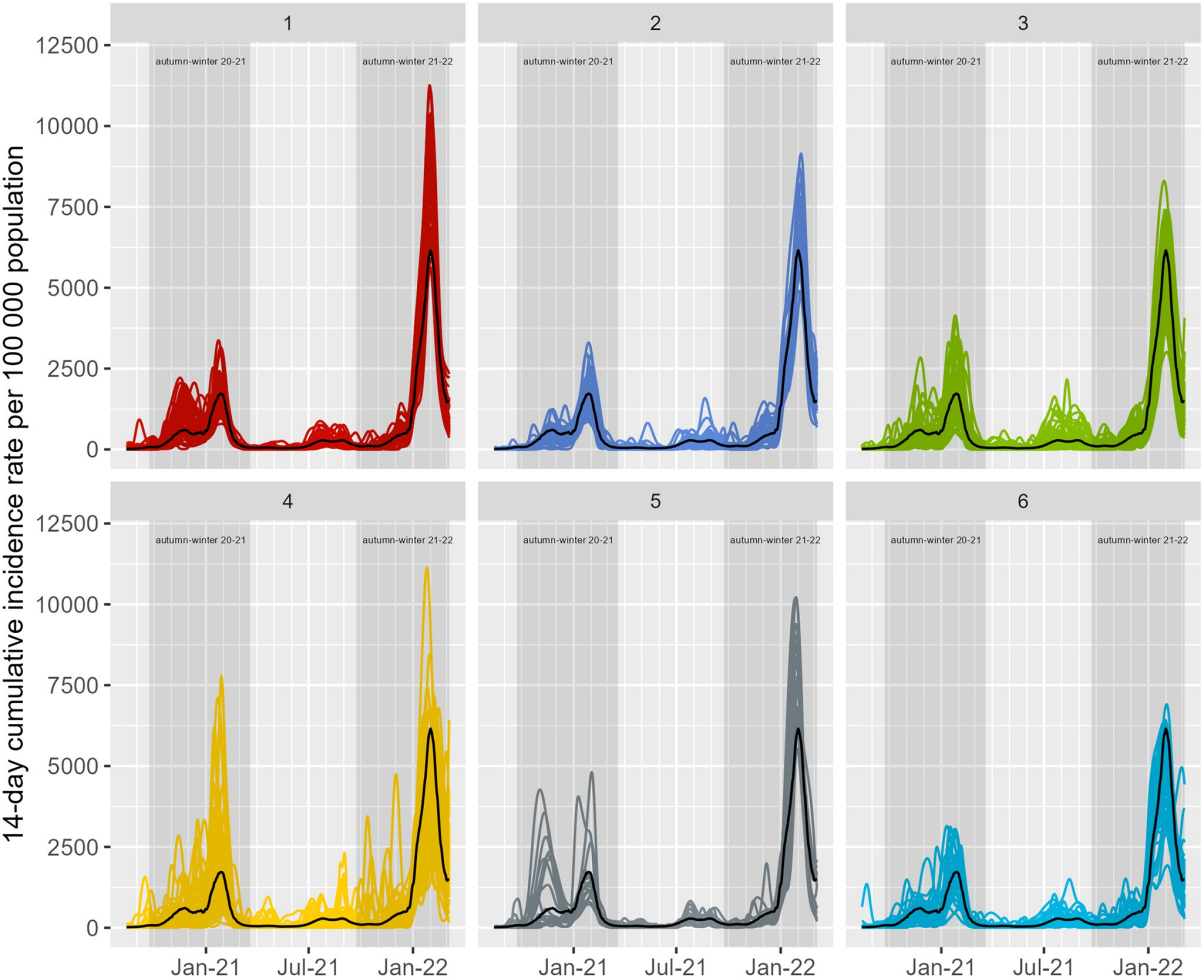
Municipality’s incidence curves by cluster. The plot shows the functional data curves of COVID-19 incidence rates by municipality from August 2020 to March 2022, for the six clusters. The line curve in black represents the mean Portuguese incidence curve.

After a classification of functional data, we found clusters of municipalities located along the northern coast and adjacent regions (cluster 1), central coast (cluster 2), hinterland regions from north to south plus southern coast (clusters 3, 4 and 5), and the metropolitan area of Lisbon plus municipalities lying deep along the hinterland (cluster 6). The curves from municipalities classified in each cluster are presented in Fig 9, along with the estimated mean national curve. The differences between clusters are mainly defined by the average height of the curves (i.e., amplitude) in the autumn-winter seasons. We note, for example, that in the autumn-winter season of 2021–2022, there is a substantial difference in the number of curves above and below the national mean curve in cluster 1 and cluster 6, respectively.

**Fig 10:**
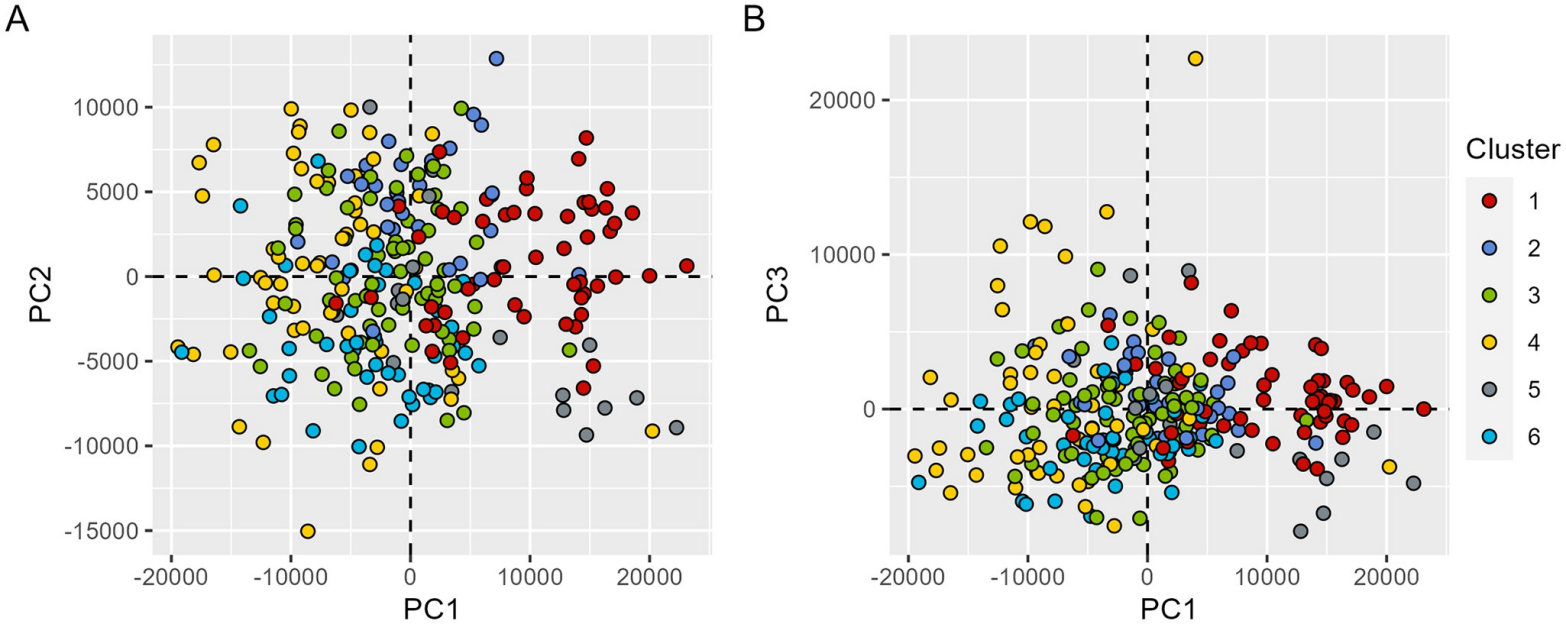
Scores of municipalities projected on the first and second functional PC (A), and first and third functional PC (B). Each point represents one municipality, and is coloured according to clustering results. See also S1 Fig for a clearer visualization of the separation between clusters.

The scatterplots of the first three principal component scores (Fig 10) illustrate the ability of the proposed method to discriminate municipalities with distinct spatiotemporal variations as revealed by incidence curves. In these scatterplots, municipalities’ scores (as in Eq 4) are classified according to the clusters returned by hierarchical clustering to facilitate the interpretation of results.

The projection of municipalities’ scores in the first two functional PCs is illustrated in Fig 10A. The first component scores (PC1) separate most of the municipalities from clusters 1 (red) and 5 (grey) with positive scores, from municipalities of clusters 4 (yellow) and 6 (light blue) with negative scores. Positive scores are associated with incidence curves above the overall (national) average incidence, while negative scores are associated with incidence curves below the overall (national) average. Most of municipalities from clusters 1 and 5 are located along the northern coast and adjacent regions, while municipalities from clusters 4 and 6 are mostly located in inland areas of Portugal (Fig 8).

The second component shows the other type of variability that splits most municipalities of cluster 2 (dark blue) with positive scores from municipalities in clusters 5 (grey) and 6 (light blue) with negative scores. This component shows that municipalities clustered in clusters 5 and 6 as the most relevant contributors towards the lower incidence rates observed along the autumn-winter periods.

**Fig 11:**
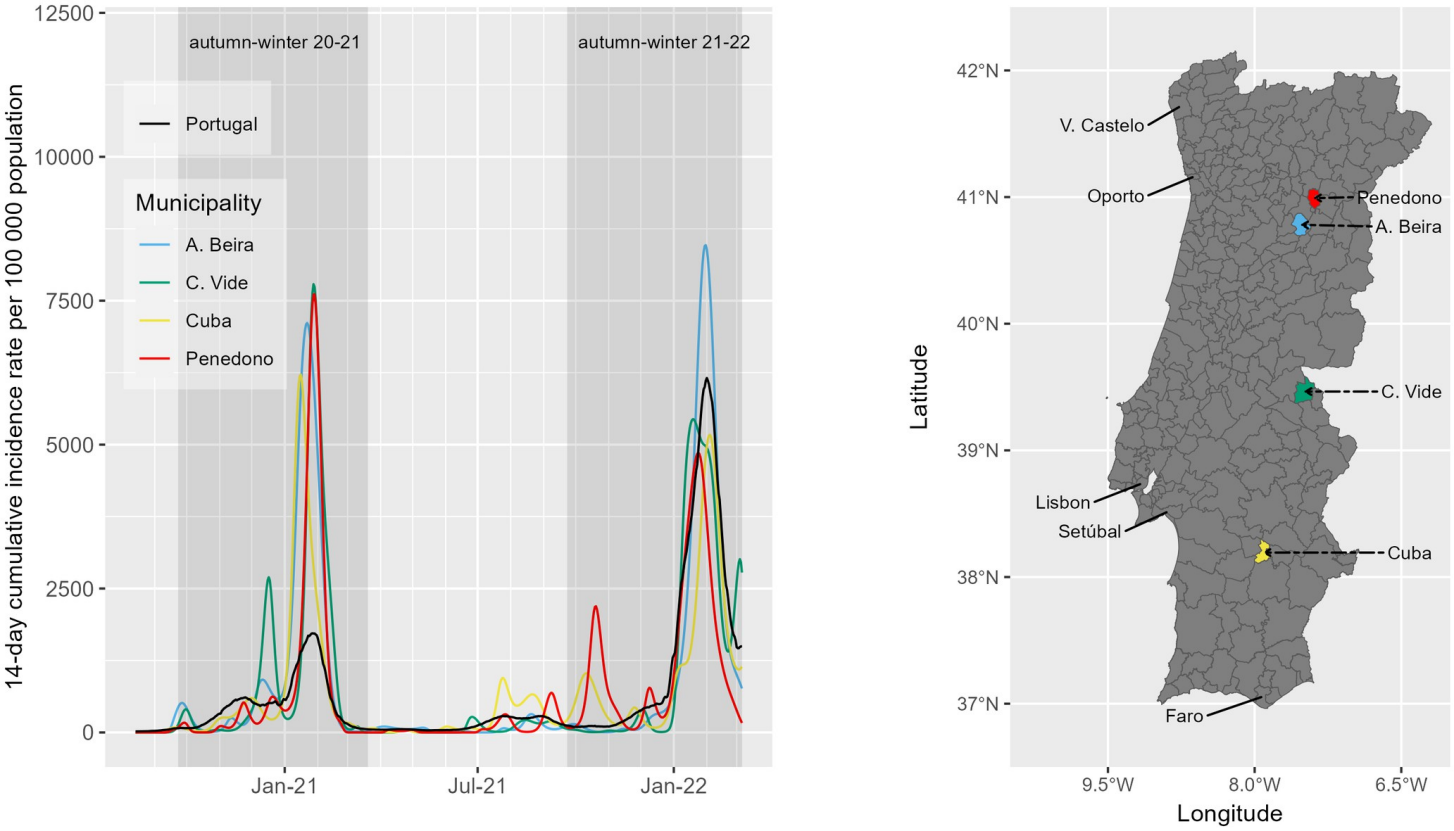
COVID-19 incidence rate curves of the four municipalities with the highest scores in functional PC3 (representing the “outbreak effect”), between August 2020 and March 2022. (A) The four municipalities belong to cluster 4. The line curve in black represents the mean Portuguese incidence curve. (B) The geographic location of the four municipalities in Portugal. The official administrative base map of Portugal is in public domain, courtesy of the Portuguese Direção-Geral do Território (https://www.dgterritorio.gov.pt/).

The position of municipalities (scores) in the third component (corresponding to the “outbreak effect”) are illustrated by Fig 10B (y-axis). It is possible to highlight four municipalities from cluster 4 with the highest scores in this component. These municipalities have small populations, and have all suffered very high incidence rates in winter 2020–2021, mainly due to outbreaks occurring in nursing homes. The incidence curves of these four municipalities are shown in Fig 11.

## Discussion

In this study, we integrate FDA with unsupervised learning and spatial statistics to develop an approach for detecting the main spatiotemporal patterns of COVID-19 incidence. As the methods used here are well established, the novelty relies on their use for an analysis of public health data. With the proposed approach, we were able to describe a coherent story of the spatiotemporal evolution of COVID-19 incidence on the Portuguese mainland at the municipality level between August 2020 and March 2022, and to identify curve patterns describing different features found in the data over time and space.

### FDA and clustering

Using FDA, we estimated not only the distribution of incidence variability in any given period of time, but we also reduced the data noise through curve smoothing. These smoothed curves were highly relevant in detecting the major patterns of variability, and a first step towards dimension reduction throughout the period analysed. By applying FPCA to the curves, we provided a means for new insights and clearer interpretations about the covariance, or correlation structures, in the spatially correlated multidimensional functional datasets (i.e., municipalities’ functional curves), reducing the interpretation of initial 278 dimensions to only 3 dimensions explaining approximately 72% of the total variability. By applying these functional approaches to the incidence rates, we provide a valid alternative solution to minimize the number of curves to be analysed while maximizing the retention of relevant information (i.e., variability) that the original data exhibited. Another positive aspect of FDA was the possibility to analyse the evolution of incidence rates over a period of 1.6 years (570 consecutive days), without separating periods with distinct evolution patterns, such as seasonality (e.g., winter vs. summer). While separating the incidence rate curves in different periods would allow a better understanding and description of particular seasons, this would limit the analysis of the pandemic as a whole.

The clustering technique used explicitly includes spatial structure dependency in the analysis. This was key in describing the major patterns of incidence curves across the country, since municipalities close to each other tend to be more similar (clustered). In fact, the approach used here (hierarchical clustering of functional data with spatial dependence) offers the possibility of combining knowledge from spatial statistics and FDA [39]. Using a trace-variogram allowed us to efficiently describe the spatial variation of incidence curves in terms of shape and range distance. Therefore, an obvious advantage of adding weights representing the spatial dependence of data to the dissimilarity matrix was the ability to obtain spatially structured clusters of municipalities.

Compared to other exploratory spatiotemporal approaches used to monitor COVID-19 distribution, one advantage of our approach is the suitable way used to describe spatial patterns in incidence variability over a continuous time domain. By modelling a trace-variogram to quantify spatial correlation and to weight the dissimilarity matrix among incidence curves, we explicitly account for spatial dependence in combination with hierarchical clustering of functional data to describe major regional patterns of incidence over time. Most exploratory spatiotemporal methods, either based on Local Indicators of Spatial Association [4, 16, 43–45] or retrospective Space-Time Scan Statistic [46–48], describe temporal patterns with time-series data ignoring the continuous smooth dynamic and noise reduction provided by functional approaches. Other works [23, 48–50] used functional data analysis and unsupervised learning methods to model and extract patterns from time-series data, showing the relevance of applying these approaches to identify temporal dynamic patterns of COVID-19. Our approach proposes a full spatiotemporal analysis describing COVID-19 patterns, which combines a comprehensive approach to extract temporal evolution/dynamic patterns with the spatial correlation analysis that describes their spatial structure. Simultaneously detecting spatial patterns of temporal COVID-19 distribution can be especially important for public health authorities to improve their risk communication strategies, tailoring messaging to specific regions or communities based on local contexts, culture and risk factors [51].

### Spatiotemporal evolution

The temporal pattern found in the first functional PC (Fig 5, PC1) matches the curve shape found for the overall mean incidence evolution in Portugal (e.g., see Fig 2), and defines the municipalities that had higher and increasingly above the mean incidence rates, especially in autumn-winter 2021–2022. The sharper increase in incidence and variability patterns observed between December 2021 and February 2022 is consistent with the dissemination of the Omicron variant [52, 53], the easing of restrictive public health measures that occurred after the long second and third waves of the COVID-19 pandemic, and the sense of safety created by the implementation of vaccination programs [32].

The vast majority of municipalities located on the northern coast and adjacent regions are those that had relatively high incidence rates in that period, and are classified in clusters 1 and 5 (Fig 8). A closer look into the shapes of the curves in these two clusters shows that they display above-average incidence rates, especially in the autumn-winter season of 2021–2022 (see Fig 9, clusters 1 and 5). The agreement between the behaviour of the incidence curves and their geographical locations appears to illustrate some unmeasured/unknown underlying processes (e.g., socio-demographic, cultural, environmental processes, previous exposure to SARS-CoV-2) taking place in that geographical area. Identifying these processes is beyond the scope of this work. Nonetheless, previous research from Almendra et al. [54] outlined a geographical pattern compatible with our results. Specifically, they found socio-demographic and economic factors to have a higher impact on COVID-19 incidence in northern regions along the Porto region and along the Atlantic shore, due to stronger cultural relationships between neighbouring municipalities. Duarte et al. [18] also identified socio-demographic and economic profiles as relevant risk factors for the increased COVID-19 incidence observed in the same region during the winter 2020–2021.

The temporal pattern found in the second PC (Fig 5, PC2) mostly represents the contribution of municipalities with lower than the mean incidence up to autumn-winter 2021–2022. The municipalities in this group are mostly classified in cluster 6, showing a spatial distribution pattern dispersed along Portugal’s hinterland plus the region of Lisbon (Fig 8). While these results are difficult to interpret due to their spatial dispersion and the small proportion of total variation contained, they provide a meaningful spatial and temporal pattern consistent with the successful adoption of preventive measures (e.g., social distancing), in particular the impact of tiered and nationwide lockdowns [32], or the impact of public health interventions [42] (e.g., vaccination).

In the third functional PC, the temporal pattern reflects the contribution of municipalities with relatively higher than the mean incidence during the autumn-winter 2020–2021 season. The most relevant contributions to this PC coincide with municipalities located in the hinterland, where population sizes are small, and COVID-19 outbreaks reported in that period (Fig 11). We believe this result illustrates the ability of this approach to identify and monitor outbreak clusters’ occurrences for health surveillance.

The use of a variogram analysis to measure spatial correlation allowed for the detection of spatially homogenous COVID-19 incidence curves. By first estimating a trace-variogram and later fitting an exponential model, we found that incidence curves are correlated up to 227 km. COVID-19 incidence is indeed known to be spatially correlated [55, 56]. Accordingly, our results show that taking into consideration spatial correlation is crucial when analysing disease incidence data. On the other hand, we found that 38% of spatial variability had no spatial pattern. This result might be regarded as variability caused by the absence of data over small distances (e.g., sparse municipalities), or by unmeasured factors [57]. For instance, incidence rate estimates are sensitive to changes in population sizes [58] and do increase spatial variability when population sizes are heterogenous or small [59, 60]. At the same time, the areas of municipalities contribute towards increasing spatial variability due to their irregular shapes, sizes and orientations [61]. By combining all these factors, we are likely looking at the main contributors to the estimated nugget-effect. Other relevant issues are those related to mobility rates [62], or testing availability, since they are expected to influence spatiotemporal patterns of incidence rates. For example, neighbour municipalities with different incidence curves and mobility dynamics could eventually be more similar if mobility and testing intensity had been included in our analysis. We must also bear in mind that the spatiotemporal patterns described are based on smoothed curves fit to the original incidence data, and require caution in interpretation. We are assuming that the features identified in our study (i.e., global effects, preventive measures and outbreak effects) are described well by the smoothed curves representing the underlying dynamics of COVID-19 incidence. Finally, the description and interpretation of principal component and hierarchical clustering is inevitably subjective, and must be regarded as exploratory [63].

As shown in our results, spatial and temporal features separate the northern and coastal regions from the southern and hinterland, and the effects in 2020–2021 from the effects in the 2021–2022 autumn-winter season. While there is no objective measurable way to validate these findings (due to the limitation of unsupervised learning methods), our results are coherent and compatible with the results from other authors. On the basis of the above, we believe that the results contribute towards characterizing the major spatiotemporal COVID-19 incidence patterns found in Portugal, using an innovative and straightforward solution. Our results can help public health authorities to identify high-risk areas, improve their risk communication strategies, adapt NPIs and allocate resources more effectively. Based on our cluster findings, public health authorities can make more informed decisions, allowing them to determine which measures are most effective, and adjust their strategies accordingly. Results can also be integrated with another data sources (e.g., related to transmissibility, vaccination, resource allocation, mobility) and transferred for disease surveillance to provide predictive analytics or prospective detection of spatiotemporal clusters.

## Conclusions

In this study, we propose a novel approach integrating FDA with unsupervised learning and spatial statistics to detect the main spatiotemporal patterns of COVID-19 incidence and applied it to the Portuguese mainland. With FDA, we were able to model the shape of Portuguese municipalities’ incidence curves and use them to extract the main temporal patterns of variability and cluster the municipalities by curve similarity, while considering their spatial proximity. We analysed a period of 1.6 years and found three main patterns of variability occurring in autumn-winter seasons in different spatial clusters of municipalities. The results for the spatiotemporal classification are coherent and compatible with results reported by other researchers, showing that the proposed approach detects the main spatiotemporal patterns of COVID-19 incidence.

A natural extension of the methods proposed here can be easily achieved by expanding the exploratory approach to the analysis of the first and second derivatives of incidence functional data (representing incidence velocity and acceleration, respectively) following similar arguments as those presented here. It is also possible to expand the approach to a functional regression modelling framework, allowing the incorporation of dynamic (e.g., mobility, testing, vaccination) and static (e.g., socio-demographic and economic) predictors in the modelling process to analyse their spatiotemporal impacts on incidence. As mentioned before, the idea of combining functional data with exploratory tools for COVID-19 analysis is not new. These have been used to identify temporal dynamic patterns [23]. But rather than assuming functional data as independent functions, our approach focuses more on the spatial nature of SARS-CoV-2 transmission and claims that spatially correlated curves should be considered instead, as they can alter the spatiotemporal configuration of COVID-19 disease patterns. Based on this, we believe that the novel approach extends and refines existing exploratory tools for analysing spatiotemporal public health data, bringing added value to the understanding on the main aspects involving the spread patterns of disease.

## Supporting information

S1 Fig Score plots of municipalities to visualize separation between clusters in functional PC1-PC2 (A) and PC1-PC3 (B). Each plot shows the scores of municipalities belonging to one cluster in a different colour (same colour scheme as presented in Figs 8–10) and the scores of the municipalities belonging to the other clusters in grey colour.
(TIF)
