# Supplementary figures and images for: Understanding spatiotemporal patterns of COVID-19 incidence in Portugal: A functional data analysis from August 2020 to March 2022
Source: PLoS One. 2024 Feb 1;19(2):e0297772. doi: 10.1371/journal.pone.0297772 (PMC10833534; doi:10.1371/journal.pone.0297772)

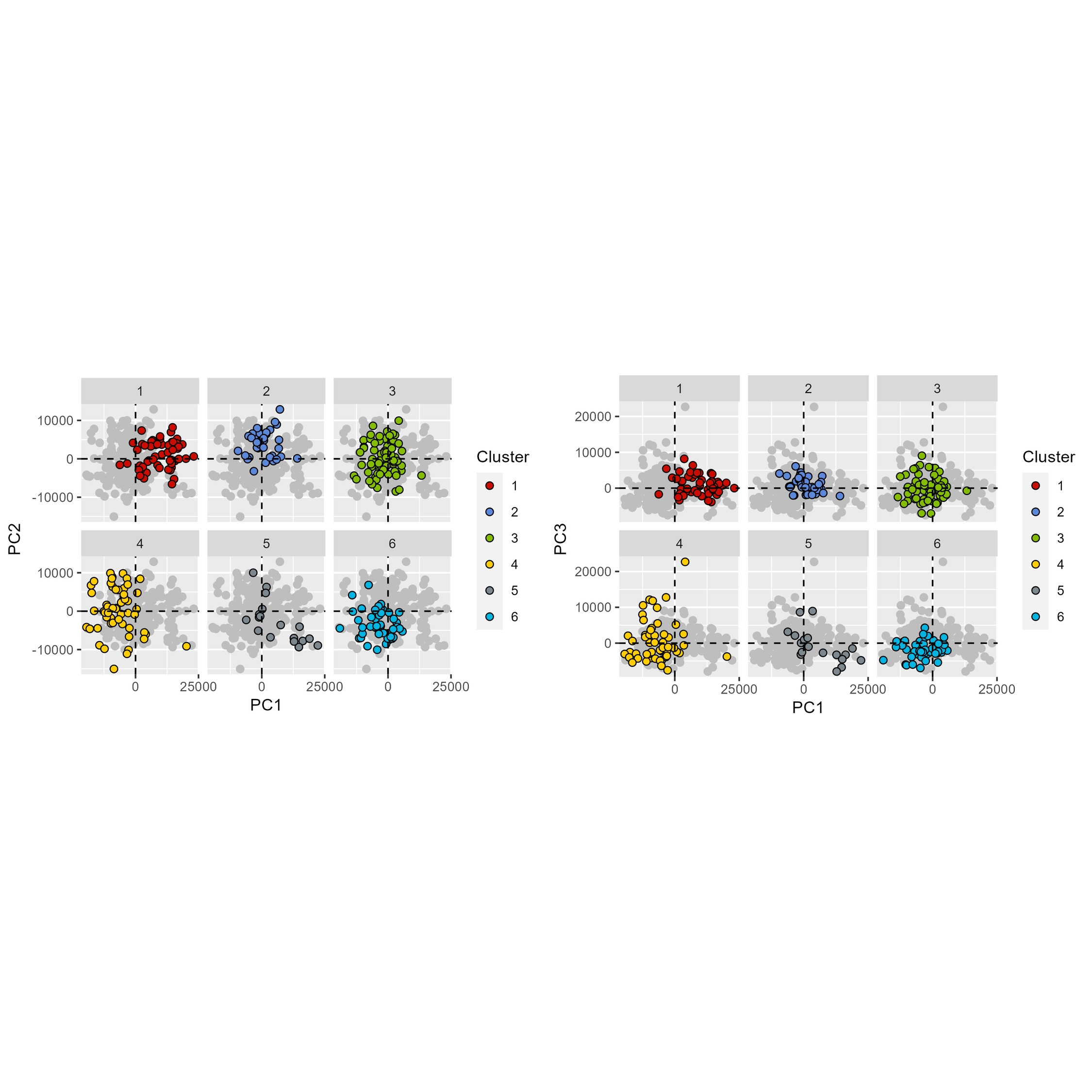

Supplement: S1 Fig — Each plot shows the scores of municipalities belonging to one cluster in a different colour (same colour scheme as presented in Figs 8–10) and the scores of the municipalities belonging to the other clusters in grey colour. (TIF) [file pone.0297772.s001.tif]
